# An Automatic Pixel-Wise Multi-Penalty Approach to Image Restoration

**DOI:** 10.3390/jimaging9110249

**Published:** 2023-11-15

**Authors:** Villiam Bortolotti, Germana Landi, Fabiana Zama

**Affiliations:** 1 Department of Civil, Chemical, Environmental, and Materials Engineering, University of Bologna, 40131 Bologna, Italy; villiam.bortolotti@unibo.it; 2 Department of Mathematics, University of Bologna, 40127 Bologna, Italy; germana.landi@unibo.it

**Keywords:** multi-penalty regularization, image restoration, uniform penalty principle

## Abstract

This work tackles the problem of image restoration, a crucial task in many fields of applied sciences, focusing on removing degradation caused by blur and noise during the acquisition process. Drawing inspiration from the multi-penalty approach based on the Uniform Penalty principle, discussed in previous work, here we develop a new image restoration model and an iterative algorithm for its effective solution. The model incorporates pixel-wise regularization terms and establishes a rule for parameter selection, aiming to restore images through the solution of a sequence of constrained optimization problems. To achieve this, we present a modified version of the Newton Projection method, adapted to multi-penalty scenarios, and prove its convergence. Numerical experiments demonstrate the efficacy of the method in eliminating noise and blur while preserving the image edges.

## 1. Introduction

Image restoration is an important task in many areas of applied sciences since digital images are frequently degraded by blur and noise during the acquisition process. Image restoration can be mathematically formulated as the linear inverse problem [1]

(1) Au+e=b

where $b\in R^{M}$ and $u\in R^{N}$, respectively, are vectorized forms of the observed $m_{x}\times m_{y}$ image and the exact $n_{x}\times n_{y}$ image to be restored, $A\in R^{M\times N}$ is the linear operator modeling the imaging system, and e represents Gaussian white noise with mean zero and standard deviation σ. The image restoration problem (1) is inherently ill-posed and regularization strategies, based on the prior information on the unknown image, are usually employed in order to effectively restore the image u from b.

In a variational framework, image restoration can be reformulated as a constrained optimization problem of the form

(2) $$\begin{array}{cc} \min_{u} & \frac{1}{2}{∥Au-b∥}^{2}+R\left(u\right)\\ s.t. & u\in \Omega \end{array}$$

whose objective function contains a $L_{2}$-based term, imposing consistency of the model with the data, and a regularization term R(u), forcing the solution to satisfy some a priori properties. Here and henceforth, the symbol ∥·∥ denotes the Euclidean norm. The constraint imposes some characteristics on the solution which are often given by the physics underlying the data acquisition process. Since image pixels are known to be nonnegative, a typical choice for Ω is the positive orthant.

The quality of the restored images strongly depends on the choice of the regularization term which, in a very general framework, can be expressed as

(3) $$R\left(u\right)=\sum_{i=1}^{p}\lambda_{i}\psi_{i}\left(u\right)$$

where the positive scalars $\lambda_{i}$ are regularization parameters and the $\psi_{i}\left(u\right)$ are regularization functions for i=1,…,p. The multi-penalty approach (3) allows to impose several regularity properties on the desired solution, however a crucial issue with its realization is the need to define reliable strategies for the choice of the regularization parameters $\lambda_{i}$, i=1,…,p.

Therefore, in the literature, the most common and famous regularization approach is single-penalty regularization, also known as Tikhonov-like regularization, which corresponds to the choice p=1:

(4) R(u)=λψ(u).

In image restoration, smooth functions based on the $L_{2}$-norm or convex nonsmooth functions like the Total Variation, the $L_{1}$ norm or the Total Generalized Variation are usually used for ψ(u) in (4) [2,3]. Even in the case p=1, the development of suitable parameter choice criteria is still an open question. The recent literature has demonstrated a growing interest in multi-penalty regularization, with a significant number of researchers focusing on scenarios involving two penalty terms. Notably, the widely-used elastic regression in Statistics serves as an example of a multi-penalty regularization technique, integrating the $L_{1}$ and $L_{2}$ penalties from the Lasso and Ridge methods. However, the majority of the literature primarily addresses the development of suitable rules for parameter selection. Lu, Pereverzev et al. [4,5] have extensively investigated two $L_{2}$-based terms, introducing a refined discrepancy principle to compute dual regularization parameters, along with its numerical implementation. The issue of parameter selection is further discussed in [6], where a generalized multi-parameter version of the L-curve criterion is proposed, and in [7], which suggests a methodology based on the GCV method. Reichel and Gazzola [8] propose regularization terms of the form

(5) $$\phi_{i}\left(u\right)={∥D_{i}u∥}^{2},\;i=1,\ldots ,p$$

where $D_{i}$ are suitable regularization matrices. They present a method to determine the regularization parameters utilizing the discrepancy principle, with a special emphasis on the case p=2. Fornasier et al. [9] proposed a modified discrepancy principle for multi-penalty regularization and provided theoretical background for this a posteriori rule. Works such as [10,11,12,13,14] also explore multi-penalty regularization for unmixing problems, employing two penalty terms based on $L_{q}$ and $L_{p}$ norms, 0≤q<2 and 2≤p<∞. The latter specifically concentrates on the $L_{1}$ and $L_{2}$ norms. The study [15] assesses two-penalty regularization, incorporating $L_{0}$ and $L_{2}$ penalty terms to tackle nonlinear ill-posed problems and analyzes its regularizing characteristics. In [16], an automated spatially adaptive regularization model combining harmonic and Total Variation (TV) terms is introduced. This model is dependent on two regularization parameters and two edge information matrices. Despite the dynamic update of the edge information matrix during iterations, the model necessitates fixed values for the regularization parameters. Calatroni et al. [17] present a space-variant generalized Gaussian regularization approach for image restoration, emphasizing its applicative potential. In [18], a multipenalty point-wise approach based on the Uniform Penalty principle is considered and analyzed for general linear inverse problems, introducing two iterative methods, UpenMM and GUpenMM, and analyzing their convergence.

Here, we extend the methodology developed in [18] to image restoration problems and we perform a comparative analysis with state-of-the-art regularization methods for this application. We propose to find an estimate $u^{*}$ of u satisfying

(6) $$\begin{array}{cc} u^{*}= & \arg\min_{u\geq 0}\;\frac{1}{2}{∥Au-b∥}^{2}+\frac{1}{2}\sum_{i=1}^{N}\lambda_{i}^{*}{\left(Lu\right)}_{i}^{2},\\ \lambda_{i}^{*}= & \frac{∥Au^{*}{-b∥}^{2}}{N({\left(Lu^{*}\right)}_{i}^{2}+\epsilon )}. \end{array}$$

where ϵ is a positive scalar and $L\in R^{N\times N}$ is the discrete Laplacian operator. This model, named MULTI, is specifically tailored for the image restoration problem. Observe that MULTI incorporates a pixel-wise regularization term and includes a rule for choosing the parameters. We formulate an iterative algorithm for computing the solution $\left(u^{*},\lambda^{*}\right)$ of (6), where $\lambda^{*}={\left(\lambda_{1}^{*},\ldots ,\lambda_{N}^{*}\right)}^{T}$. Once the regularization parameters are set in every inner iteration, the constrained minimization subproblem is efficiently solved by a customized version of the Newton Projection (NP) method. Here, the Hessian matrix is approximated by a Block Circulant with Circulant Blocks (BCCB) matrix, which is easily invertible in the Fourier space. This modified version of NP was designed in [19] for single-penalty image restoration under Poisson noise and it is adapted here to the context of multi-penalty regularization. Consequently, the convergence of the modified NP method can be established.

The principal contributions of this work are summarized as follows:

1. We propose a variational pixel-wise regularization model tailored for image restoration and derived from the theoretical model developed in [18].
2. We devise an algorithm capable of effectively and efficiently solving the proposed model.
3. Through numerical experiments, we demonstrate that the proposed approach can proficiently eliminate noise and blur in smooth areas of an image while preserving its edges.

The structure of this paper is as follows: Section 2 introduces the proposed algorithm. The results of numerical experiments are presented in Section 3, and finally, the conclusions are drawn in Section 4.

## 2. Materials and Methods

In this section, we present the iterative algorithm that generates the sequence $\left(u^{\left(k\right)},\lambda^{\left(k\right)}\right)$ converging to the solution $\left(u^{*},\lambda^{*}\right)$ in (6).

Starting from an initial guess $u^{\left(0\right)}$ taken as the observed image b, the correspondent initial guess of the regularization parameters is computed as:

(7) $$\lambda_{i}^{\left(0\right)}=\frac{∥Au^{\left(0\right)}{-b∥}^{2}}{N(S_{i}^{\left(0\right)}+\epsilon )},\;i=1,\ldots ,N$$

where

(8) $$S_{i}^{\left(0\right)}=\max_{i\in N_{i}}{\left(Lu^{\left(0\right)}\right)}_{i}^{2},\;i=1,\ldots ,N$$

and $N_{i}$ is a neighborhood of size R×R, (with *R* odd and R≥1) of the i-th pixel with coordinates $\left(x_{i},y_{i}\right)$.

The successive terms $\left(u^{\left(k+1\right)},\lambda^{\left(k+1\right)}\right)$ are obtained by the update formulas reported in steps 3–5 of Algorithm 1. The iterations are stopped when the relative distance between two successive regularization vectors is smaller than a fixed tolerance Tol>0.
**Algorithm 1** Input: $\lambda^{\left(0\right)}\in R^{N}$, Tol, A,b,ϵ, Output: $\left(u^{*},\lambda^{*}\right)$1:Set k=0.2:**repeat**3:   $u^{\left(k+1\right)}=\arg\min_{u\in \Omega }\;\left\{\frac{1}{2}{∥Au-b∥}^{2}+\sum_{i=1}^{N}\lambda_{i}^{\left(k\right)}{\left(Lu\right)}_{i}^{2}\right\}$4:   $S_{i}^{\left(k+1\right)}=\max_{i\in N_{i}}{\left(Lu^{\left(k+1\right)}\right)}_{i}^{2}),\;i=1,\ldots N$5:    $\lambda_{i}^{\left(k+1\right)}=\frac{∥Au^{\left(k+1\right)}{-b∥}^{2}}{N(S_{i}^{\left(k+1\right)}+\epsilon )},\;i=1,\ldots ,N$6:   k=k+17:**until**$∥\lambda^{\left(k\right)}-\lambda^{\left(k-1\right)}∥\leq Tol∥\lambda^{\left(k\right)}∥$8:$\lambda^{*}=\lambda^{\left(k\right)}$, $u^{*}=u^{\left(k\right)}$

Algorithm 1 is well defined, and we experimentally observe a converging behaviour. Its formal convergence proof is obtained in [18] (theorem 3.4) for the case R=1, since in this case, Algorithm 1 corresponds to UPenMM. Otherwise, to preserve convergence, we should introduce a correction as proposed in the Generalized Uniform Penalty method (GUPenMM) [18]. However, even without this correction, we obtained good-quality results and we prefer here to investigate Algorithm 1 because, in the case of large scale image restoration problems, it is much more convenient from a computational point of view. Moreover, we verified that the results obtained with such a correction are qualitatively comparable with those given by Algorithm 1, as the human eye cannot distinguish differences smaller than a few gray levels.

At each inner iteration, the constrained minimization subproblem (step 3 in Algorithm 1) is solved efficiently by a tailored version of the NP method where the Hessian matrix is approximated by a BCCB matrix easily invertible in the Fourier space.

Let us denote by $J^{\left(k\right)}\left(u\right)$ the function to be minimized at step 3 in Algorithm 1:

$$J^{\left(k\right)}\left(u\right)=\frac{1}{2}{∥Au-b∥}^{2}+\sum_{i=1}^{N}\lambda_{i}^{\left(k\right)}{\left(Lu\right)}_{i}^{2}$$

and by g its gradient $\nabla J^{\left(k\right)}\left(u\right)$ where the iteration index *k* has been omitted for easier notation. Moreover, let $g_{I}$ denote the reduced gradient:

(9) $${\left\{g_{I}\right\}}_{i}=\left\{\begin{array}{cc} g_{i}, & \mathrm{if}\;i\notin I(u);\\ 0, & \mathrm{otherwise}; \end{array}\right.$$

where I(u) is the set of indices [20]:

$$I\left(u\right)=\left\{i\;|\;0\leq u_{i}\leq \varepsilon \;\mathrm{and}\;g_{i}>0\right\}$$

with

$$\varepsilon =\min\left\{\bar{\varepsilon },w\right\},\;w=∥u-{\left[u-\nabla J\left(u\right)\right]}^{+}∥$$

and $\bar{\varepsilon }$ is a small positive parameter.

The Hessian matrix $\nabla^{2}J\left(u\right)$ has the form

$$\nabla^{2}J^{\left(k\right)}\left(u\right)=A^{T}A+L^{T}\Lambda^{\left(k\right)}L$$

where $\Lambda^{\left(k\right)}$ is the diagonal matrix with diagonal elements $\lambda_{1}^{\left(k\right)},\ldots ,\lambda_{N}^{\left(k\right)}$.

A general iteration of the proposed NP-like method has the form:

(10) $$u^{\left(\ell +1\right)}={\left[u^{\left(\ell \right)}-\alpha^{\left(\ell \right)}p^{\left(\ell \right)}\right]}^{+}$$

where $p^{\left(\ell \right)}$ is the search direction, $\alpha^{\left(\ell \right)}$ is the steplength and ${\left[\cdot \right]}^{+}$ denotes the projection on the positive orthant.

At each iteration *ℓ*, the computation of $p^{\left(\ell \right)}$ requires the solution of the linear system

(11) $$H^{\left(k\right)}d^{\left(\ell \right)}=g_{I}^{\left(\ell \right)},$$

where $H^{\left(k\right)}$ is the following approximation to $\nabla^{2}J^{\left(k\right)}\left(u\right)$

(12) $$H^{\left(k\right)}=A^{T}A+\mu^{\left(k\right)}L^{T}L,\;\mu^{\left(k\right)}=\mathrm{mean}\left(\lambda_{1}^{\left(k\right)},\ldots ,\lambda_{N}^{\left(k\right)}\right).$$

Under periodic boundary conditions, $H^{\left(k\right)}$ is a BCCB matrix and system (11) can be efficiently solved in the Fourier space by using Fast Fourier Transforms. Therefore, despite its simplicity, the BCCB approximation $H^{\left(k\right)}$ is efficient, since it allows to solve the linear system in $O(N\log_{2}N)$ operations, and effective, as is shown by the numerical results. Finally, given the solution $d^{\left(\ell \right)}$ of (11), the search direction $p^{\left(\ell \right)}$ is obtained as

(13) $$p_{i}^{\left(\ell \right)}=\left\{\begin{array}{cc} d_{i}^{\left(\ell \right)}, & \mathrm{if}\;i\notin I(u^{\left(\ell \right)});\\ g_{i}^{\left(\ell \right)}, & \mathrm{otherwise}; \end{array}\right.\;i=1,\ldots ,N.$$

The step length $\alpha^{\left(\ell \right)}$ is computed with the variation of the Armijo rule discussed in [20] as the first number of the sequence ${\left\{\beta^{m}\right\}}_{m\in N}$, 0<β<1, such that

(14) $$J\left(u^{\left(\ell \right)}\right)-J\left(u^{\left(\ell \right)}\left(\beta^{m}\right)\right)\geq \eta \left(\beta^{m}\sum_{i\notin I(u^{\left(\ell \right)})}g_{i}^{\left(\ell \right)}p_{i}^{\left(\ell \right)}+\sum_{i\in I(u^{\left(\ell \right)})}g_{i}^{\left(\ell \right)}\left(u_{i}^{\left(\ell \right)}-u_{i}^{\left(\ell \right)}\left(\beta^{m}\right)\right)\right)$$

where $u^{\left(\ell \right)}\left(\beta^{m}\right)={\left[u^{\left(\ell \right)}-\beta^{m}u^{\left(\ell \right)}\right]}^{+}$ and $\eta \in (0,\frac{1}{2})$.

We observe that the approximated Hessian $H^{\left(k\right)}$ is constant for each inner iteration *ℓ* and it is positive definite, then it satisfies

$$c_{1}{∥y∥}^{2}\leq y^{T}{\left(H^{\left(k\right)}\right)}^{-1}y\leq c_{2}{∥y∥}^{2}\;\forall y\in R^{N},\forall \ell >0.$$

Following that, the results given in [19] for single-penalty image restoration under Poisson noise can be applied here to prove the convergence of the NL-like iterations to critical points.

The stopping criteria for the NP-like method are based on the relative distance between two successive iterates and the relative projected gradient norm. In addition, a maximum number of NP iterations have been fixed.

## 3. Numerical Experiments

All the experiments were performed under Windows 10 and MATLAB R2021a running on a desktop (Intel(R) Core(TM) i5-8250CPU@1.60 GHz). Quantitatively, we evaluated the quality of image restoration by the relative error (RE), improved signal to noise ratio (ISNR), and mean structural similarity index (MSSIM) measures. The MSSIM is defined by Wang et al. [21] and ISNR is calculated as:

$$\mathrm{ISNR}=20\log_{10}\left(\frac{∥b-u∥}{∥\hat{u}-u∥}\right)$$

where $\hat{u}$ is the restored image, u is the reference image, and b is the blurred, noisy image.

**Figure 1:**
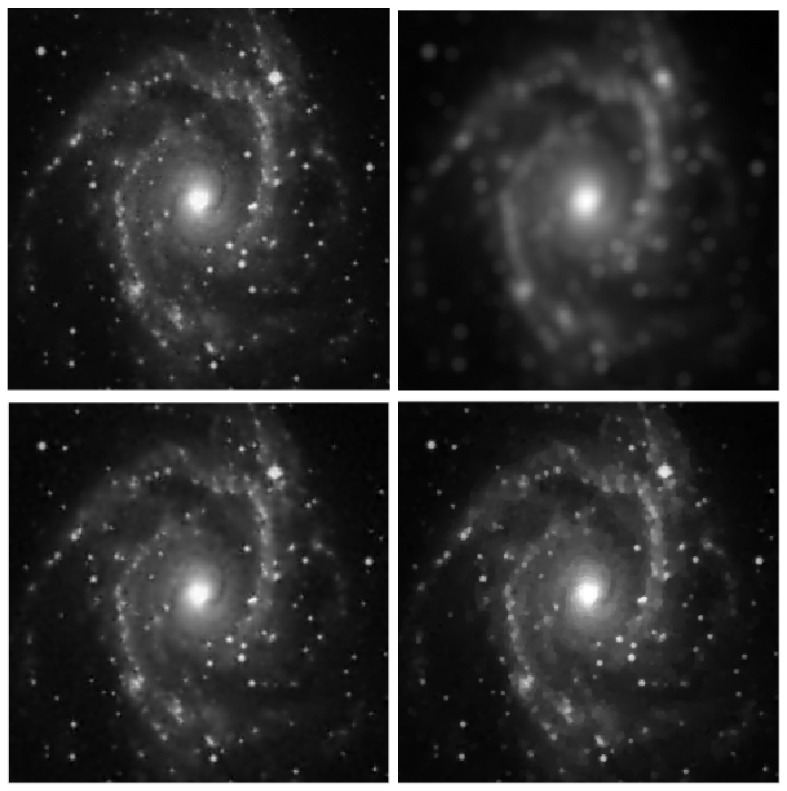
galaxy test problem: out-of-focus blur; $\delta =10^{-2}$. **Top** row: original (**left**) and blurred (**right**) images. **Bottom** row: MULTI (**left**) and TGV (**right**) restorations.

**Figure 2:**
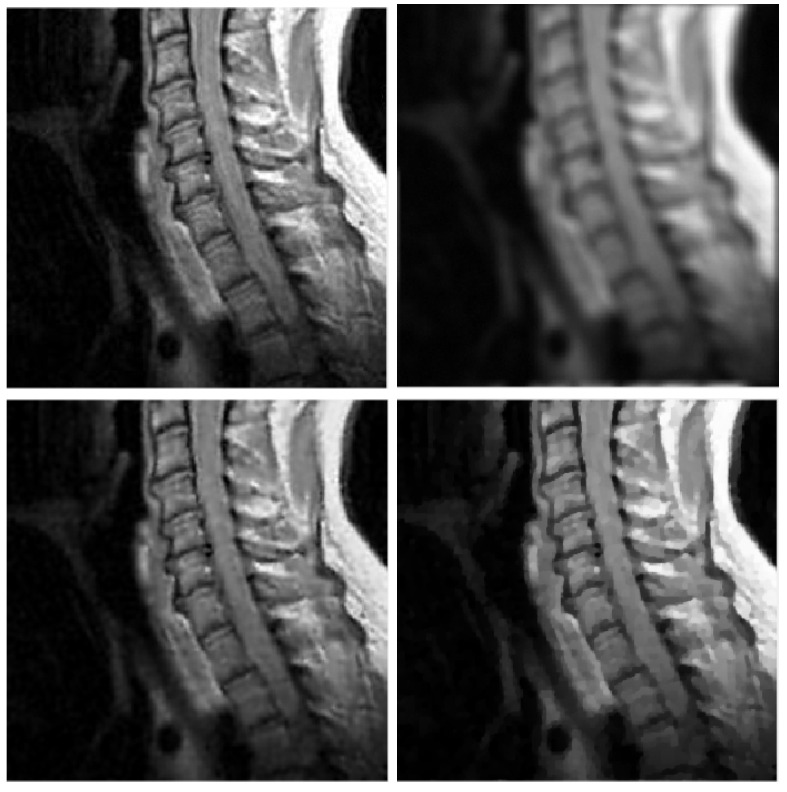
mri test problem: out-of-focus blur; $\delta =10^{-2}$. **Top** row: original (**left**) and blurred (**right**) images. **Bottom** row: MULTI (**left**) and TGV (**right**) restorations.

**Figure 3:**
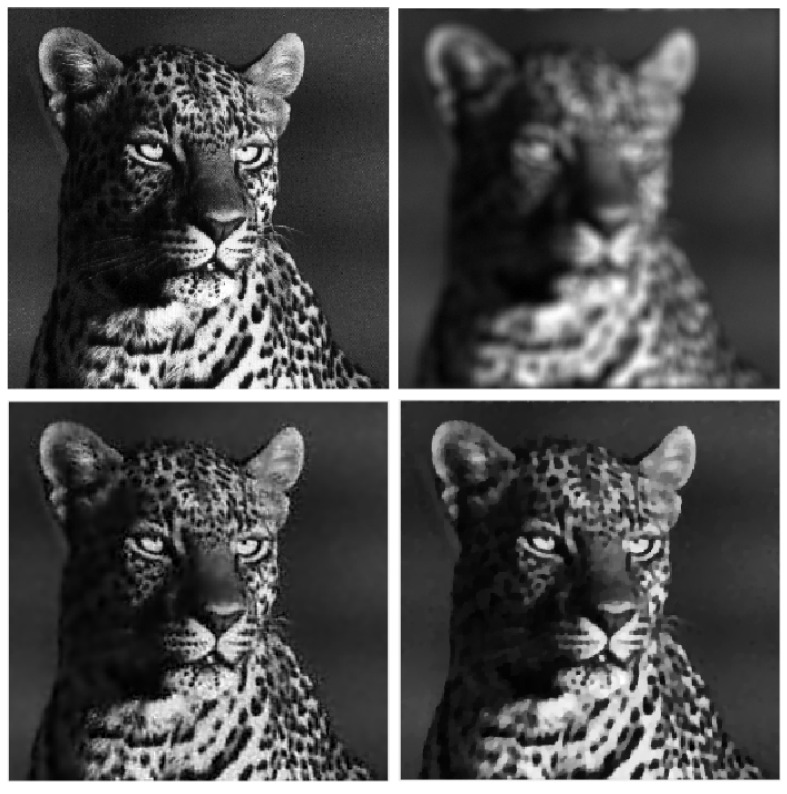
leopard test problem: out-of-focus blur; $\delta =10^{-2}$. **Top** row: original (**left**) and blurred (**right**) images. **Bottom** row: MULTI (**left**) and TGV (**right**) restorations.

**Figure 4:**
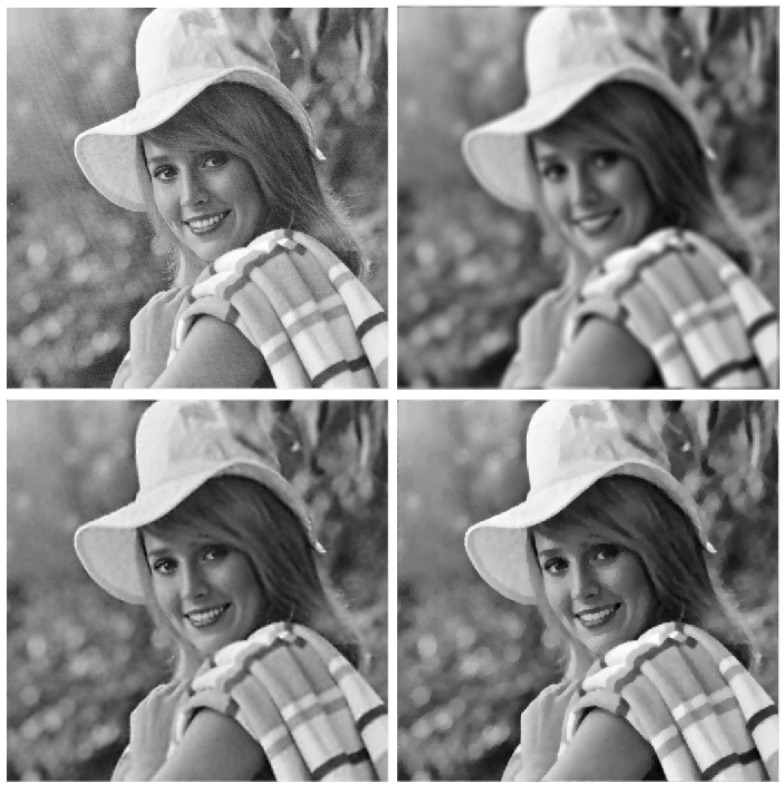
elaine test problem: out-of-focus blur; $\delta =10^{-2}$. **Top** row: original (**left**) and blurred (**right**) images. **Bottom** row: MULTI (**left**) and TGV (**right**) restorations.

Four reference images were used in the experiments: galaxy, mri, leopard, and elaine, shown in Figure 1, Figure 2, Figure 3 and Figure 4. The first three images have size 256×256, while the elaine image is 512×512. In order to define the test problems, each reference image was convolved with two PSFs corresponding to a Gaussian blur with variance 2, generated by the psfGauss function from the MATLAB toolbox Restore-Tool [1], and an out-of-focus blur with radius 5, obtained with the function fspecial from the MATLAB Image Processing Toolbox. The resulting blurred image was then corrupted by Gaussian noise with different values of the noise level δ=∥e∥/∥b∥. The values $\delta =2.5\times 10^{-2},10^{-2},5\times 10^{-3}$ were used.

We compared the proposed pixel-wise multi-penalty regularization model (MULTI) with some commonly used state-of-the-art methods based on a variational approach. In particular, we considered the Tikhonov method (TIKH) [22], the Total Variation (TV) [2], and Total Generalized Variation (TGV) [3] regularization with nonnegative constraints. Tikhonov and TV regularization are quite classic regularization terms. It is well known that Tikhonov regularization tends to make images overly smooth and it fails to preserve sharp edges. On the contrary, TV regularization better preserves sharp edges but often produces staircase effects. TGV has been recently proposed to overcome the drawbacks of Tikhonov and TV regularization such as blurring and the staircasing effect. Therefore, we compared MULTI with TGV in order to demonstrate the capacity of MULTI to preserve sharp features as well as smooth transition variations.

In our numerical experiments, the regularization parameter values for TIKH, TV, and TGV were chosen heuristically by minimizing the relative error values. The Alternating Direction Method of Multipliers (ADMM) was used for the solution of the TV-based minimization problem, while for TIKH, we used the Scaled Gradient Projection (SGP) method with Barzilai and Borwein rules for the step length selection [23]. Regarding the TGV regularization, the RESPOND method [24] was used. We remark that RESPOND has been originally proposed for the restoration of images corrupted by Poisson noise by using Directional Total Generalized Variation regularization. It has been adapted here to deal with TGV-based restoration of images under Gaussian noise. The MATLAB implementation for Poisson noise is available on GitHub at the url https://github.com/diserafi/respond (accessed on 18 September 20).

The tolerance Tol, in the outer loop of MULTI in Algorithm 1 step 7, was $10^{-1}$, while the maximum number of iterations was 20. Regarding the NP method, a tolerance of $10^{-5}$ was used and the maximum number of iterations was 1000.

The size of the neighborhood $N_{i}$ in (8) was 5×5 pixels for all tests except for galaxy, where a 3×3 neighborhood was used.

The values of the parameter ϵ in (6), used in the various tests, are in the range $\left[10^{-4},10^{-3}\right]$. In order to compare all the algorithms at their best performance, the values ϵ used in each test are reported in Table 1, where we observe that the value of ϵ is proportional to the noise level. The parameter ϵ represents a threshold and, in general, should have a small value when compared to the non-null values of $S_{i}$. We note that at the cost of adjusting a single parameter ϵ>0, it is possible to achieve point-wise optimal regularization.

Table 2, Table 3, Table 4 and Table 5 report the numerical results for all the test problems. The last column of the Tables shows the used values of the regularization parameter for TIKH, TV, and TGV while, for MULTI, it reports the norm of the regularization parameters vector $\left(\lambda_{1},\ldots ,\lambda_{N}\right)$ computed by Algorithm 1. Column 7 shows the number of RESPOND, ADMM, and SGP iterations for TGV, TV, and TIKH, respectively. For the MULTI algorithm, Column 7 shows the number of outer iterations and NP iterations in parenthesis.

In Table 2, Table 3, Table 4 and Table 5, we note that for the case of Gaussian blur, MULTI consistently achieves the best results, as highlighted in bold. This is evident from its higher MSSIM and ISNR values and lower RE values. However, for the Out-of-focus case, there are three instances (representing 25%) where TGV exhibits superior error parameters. Furthermore, our observations indicate that TGV consistently outperforms both TIKH and TV in terms of accuracy. Therefore, in Figure 1, Figure 2, Figure 3 and Figure 4 we only represent the images obtained by MULTI and TGV in the out-of-focus case, with $\delta =10^{-2}$, as this is a very challenging case.

**Figure 5:**
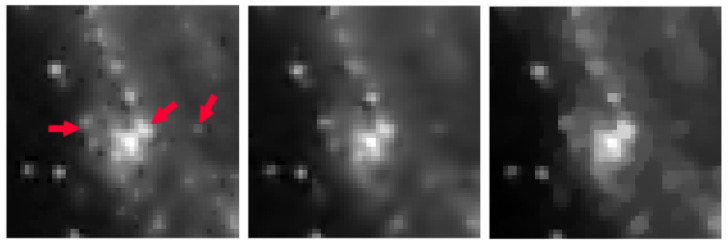
galaxy test problem: out-of-focus blur; $\delta =10^{-2}$. A detail of the original image (**left**), MULTI restoration (**centre**), and TGV restoration (**right**). Red arrows highlight the different image features.

**Figure 6:**
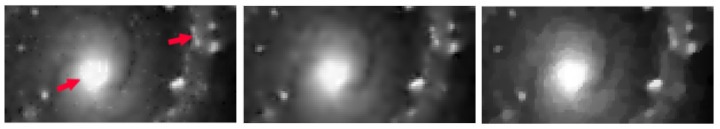
galaxy test problem: out-of-focus blur; $\delta =10^{-2}$. A detail of the original image (**left**), MULTI restoration (**centre**), and TGV restoration (**right**). Red arrows highlight the different image features.

**Figure 7:**
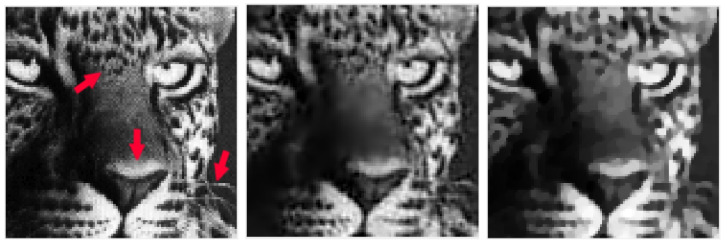
leopard test problem: out-of-focus blur; $\delta =10^{-2}$. A detail of the original image (**left**), MULTI restoration (**centre**), and TGV restoration (**right**). Red arrows highlight the different image features.

**Figure 8:**
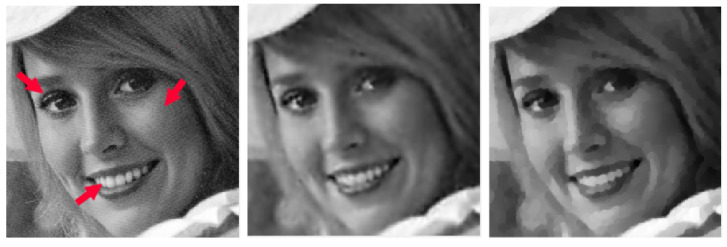
elaine test problem: out-of-focus blur; $\delta =10^{-2}$. A detail of the original image (**left**), MULTI restoration (**centre**), and TGV restoration (**right**). Red arrows highlight the different image features.

The strength of MULTI is evident by observing some details of the reconstructed images. In Figure 5, Figure 6, Figure 7 and Figure 8 we show some cropped details of the original images and compare it with MULTI and TGV reconstructions. Figure 5 shows a detail of the galaxy with a few stars over a dark background. In this case the image sparsity is better preserved by MULTI. Figure 6 shows the galaxy centre: it is a smooth area which is well recovered by MULTI while TGV shows staircasing. In Figure 7 we observe that the leopard’s whiskers and fur spots are better reproduced by MULTI. Moreover, from the images provided in Figure 8, it can be observed that MULTI method better preserves the local characteristics of the image, avoiding flattening the smooth areas and optimally preserving the sharp contours. We observe that a smooth area such as the cheek is better represented by MULTI, avoiding the staircase effect. Moreover, an area with strong contours, such as the teeth and the eyes, is better depicted. In summary, these examples show the good capacity of MULTI to preserve the different image structures, narrow peaks, and smooth areas by using local regularization parameters that are inversely proportional to the local curvature approximated by the discrete Laplacian.

**Figure 9:**
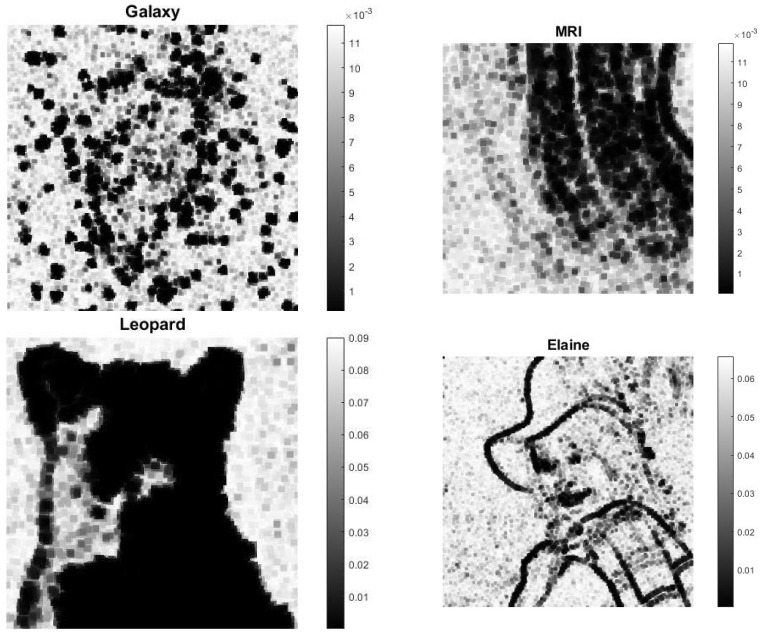
Computed regularization parameters: out-of-focus blur, $\delta =10^{-2}$.

The regularization parameters computed by MULTI are represented in Figure 9; the adherence of the regularization parameters’ values to the image content is clear, showing larger values in areas where the image is flat and smaller values where there are pronounced gradients (edges). The range of the parameters automatically adjusts according to the different test types.

**Figure 10:**
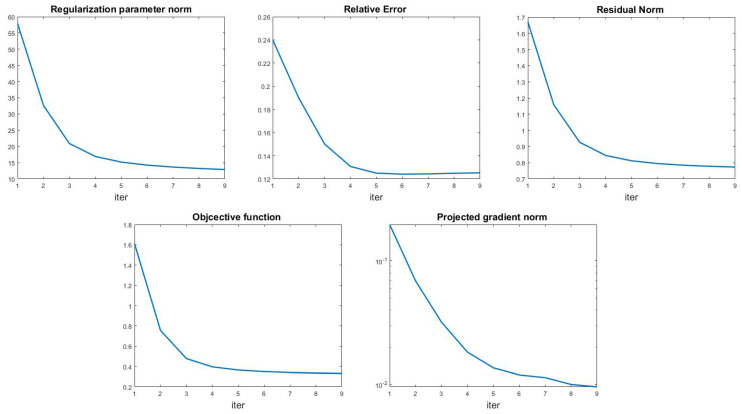
Leopard test problem (out-of-focus blur, $\delta =10^{-2}$). Top line: regularization parameters norm (**left**), relative error (**middle**), and residual norm (**right**) history for the multi-penalty model. Bottom line: objective function (**left**) and projected gradient norm history (**right**).

Finally, we show in Figure 10 an example of the algorithm behavior, reporting the history of the regularization parameter norm, relative error, and residual norm (top row), in the case of leopard test with out-of-focus blur and noise level $\delta =10^{-2}$. In the bottom row we show the decreasing behavior of the objective function and projected gradient norm. The relative error flattens after a few iterations, and the same behavior can be observed in all the other tests. Therefore, we used a large tolerance value ($Tol=10^{-1}$) in the outer loop of Algorithm 1, making it possible to obtain good regularization parameters and accurate restorations in a few outer iterations. We observe that even in the most difficult case, Table 4 row 12, the total computation time is 285 s, proving the algorithm efficiency.

## 4. Conclusions

Despite the interest of recent literature on multi-penalty regularization, its drawback lies in the difficult computation of the regularization parameters. Our work proposes the pixel-wise regularization model to tackle the significant task of image restoration, concentrating on eliminating degradation originating from blur and noise. We show that multi-penalty regularization can be realized by an algorithm that is able to compute efficiently and automatically a large number of regularization parameters. The numerical results confirm the algorithm’s proficiency in eliminating noise and blur while concurrently preserving the edges of the image. Such an approach can be exploited in different real-world imaging applications, such as computed tomography, super-resolution, and biomedical imaging in general. Finally, further analyses of the properties of the proposed algorithm will be the subject of future works.

## Figures and Tables

**Table 1:** Values for the parameter ϵ in (7).

Test Problem	Blur	$\delta =2.5\times 10^{-2}$	$\delta =10^{-2}$	$\delta =5\times 10^{-3}$
galaxy	Out-of-focus	0.5 × 10${}^{-3}$	0.25 × 10${}^{-3}$	0.1 × 10${}^{-3}$
	Gaussian	0.5 × 10${}^{-3}$	0.25 × 10${}^{-3}$	0.1 × 10${}^{-3}$
mri	Out-of-focus	1.5 × 10${}^{-3}$	1 × 10${}^{-3}$	0.5 × 10${}^{-3}$
	Gaussian	1.5 × 10${}^{-3}$	1 × 10${}^{-3}$	0.5 × 10${}^{-3}$
leopard	Out-of-focus	2.5 × 10${}^{-3}$	1.5 × 10${}^{-3}$	1. × 10${}^{-3}$
	Gaussian	2.5 × 10${}^{-3}$	0.1 × 10${}^{-3}$	0.5 × 10${}^{-4}$
elaine	Out-of-focus	1 × 10${}^{-3}$	1 × 10${}^{-3}$	1 × 10${}^{-3}$
	Gaussian	1 × 10${}^{-3}$	0.5 × 10${}^{-3}$	0.5 × 10${}^{-3}$

**Table 2:** Numerical results for the galaxy test problem. Column Iters shows the number of RESPOND, ADMM and SGP iterations for TGV, TV and TIKH, respectively. For the MULTI algorithm, it shows the number of outer iterations and NP iterations in parenthesis. The best results are highlighted in bold. Column λ shows the used values of the regularization parameter for TIKH, TV and TGV while, for MULTI, it reports the norm of the regularization parameters vector $\left(\lambda_{1},\ldots ,\lambda_{N}\right)$ computed by Algorithm 1.

Blur	δ	Model	RE	ISNR	MSSIM	Iters	λ
Out-of-focus	$2.5\times 10^{-2}$	TGV	9.5953 × 10${}^{-2}$	7.2175 × 10${}^{0}$	9.1418 × 10${}^{-1}$	226	3.0000 × 10${}^{2}$
		TV	1.0268 × 10${}^{-1}$	6.6291 × 10${}^{0}$	8.7089 × 10${}^{-1}$	278	1.0000 × 10${}^{-4}$
		TIKH	1.3864 × 10${}^{-1}$	4.0211 × 10${}^{0}$	8.3486 × 10${}^{-1}$	200	1.1000 × 10${}^{-2}$
		MULTI	**8.2096 × 10${}^{-2}$**	**8.5722 × 10${}^{0}$**	**9.3431 × 10${}^{-1}$**	4(857)	8.4116 × 10${}^{0}$
	$10^{-2}$	TGV	7.1519 × 10${}^{-2}$	9.7292 × 10${}^{0}$	9.5015 × 10${}^{-1}$	302	7.5000 × 10${}^{2}$
		TV	6.7196 × 10${}^{-2}$	1.0271 × 10${}^{1}$	9.4744 × 10${}^{-1}$	259	5.0000 × 10${}^{-5}$
		TIKH	1.0943 × 10${}^{-1}$	6.0351 × 10${}^{0}$	8.5965 × 10${}^{-1}$	200	3.0000 × 10${}^{-3}$
		MULTI	**6.2660 × 10${}^{-2}$**	**1.0878 × 10${}^{1}$**	**9.5843 × 10${}^{-1}$**	7(1061)	2.1493 × 10${}^{0}$
	$5\times 10^{-3}$	TGV	6.1028 × 10${}^{-2}$	1.1102 × 10${}^{1}$	9.6274 × 10${}^{-1}$	323	1.0000 × 10${}^{3}$
		TV	6.3776 × 10${}^{-2}$	1.0719 × 10${}^{1}$	9.4229 × 10${}^{-1}$	303	1.0000 × 10${}^{-5}$
		TIKH	9.1181 × 10${}^{-2}$	7.6143 × 10${}^{0}$	8.7665 × 10${}^{-1}$	200	1.0000 × 10${}^{-3}$
		MULTI	**4.8955 × 10${}^{-2}$**	**1.3017 × 10${}^{1}$**	9.7150 × 10${}^{-1}$	9(1013)	1.0491 × 10${}^{0}$
Gaussian	$2.5\times 10^{-2}$	TGV	9.6150 × 10${}^{-2}$	4.5854 × 10${}^{0}$	9.3328 × 10${}^{-1}$	198	2.5000 × 10${}^{2}$
		TV	8.8306 × 10${}^{-2}$	5.3246 × 10${}^{0}$	9.2702 × 10${}^{-1}$	224	1.0000 × 10${}^{-4}$
		TIKH	1.0047 × 10${}^{-1}$	4.2032 × 10${}^{0}$	9.0136 × 10${}^{-1}$	200	5.0000 × 10${}^{-3}$
		MULTI	**7.3686 × 10${}^{-2}$**	**6.8966 × 10${}^{0}$**	**9.5114 × 10${}^{-1}$**	4(699)	8.3317 × 10${}^{0}$
	$10^{-2}$	TGV	8.5737 × 10${}^{-2}$	5.5019 × 10${}^{0}$	9.4922 × 10${}^{-1}$	246	1.0000 × 10${}^{3}$
		TV	7.7929 × 10${}^{-2}$	6.3312 × 10${}^{0}$	9.3945 × 10${}^{-1}$	223	1.0000 × 10${}^{-5}$
		TIKH	8.4284 × 10${}^{-2}$	5.6503 × 10${}^{0}$	9.2110 × 10${}^{-1}$	200	1.0000 × 10${}^{-3}$
		MULTI	**6.0402 × 10${}^{-2}$**	**8.5442 × 10${}^{0}$**	**9.6606 × 10${}^{-1}$**	5(437)	2.3968 × 10${}^{0}$
	$5\times 10^{-3}$	TGV	8.0734 × 10${}^{-2}$	6.0131 × 10${}^{0}$	9.5637 × 10${}^{-1}$	280	2.5000 × 10${}^{3}$
		TV	7.3912 × 10${}^{-2}$	6.7800 × 10${}^{0}$	9.5186 × 10${}^{-1}$	214	1.0000 × 10${}^{-6}$
		TIKH	7.3620 × 10${}^{-2}$	6.8143 × 10${}^{0}$	9.4621 × 10${}^{-1}$	200	5.0000 × 10${}^{-4}$
		MULTI	**5.7592 × 10${}^{-2}$**	**8.9471 × 10${}^{0}$**	**9.7129 × 10${}^{-1}$**	6(383)	1.3106 × 10${}^{0}$

**Table 3:** Numerical results for the mri test problem. Column Iters shows the number of RESPOND, ADMM, and SGP iterations for TGV, TV, and TIKH, respectively. For the MULTI algorithm, it shows the number of outer iterations and NP iterations in parenthesis. Column λ shows the used values of the regularization parameter for TIKH, TV, and TGV while, for MULTI, it reports the norm of the regularization parameters vector $\left(\lambda_{1},\ldots ,\lambda_{N}\right)$ computed by Algorithm 1. The best results are highlighted in bold.

Blur	δ	Model	RE	ISNR	MSSIM	Iters	λ
Out-of-focus	$2.5\times 10^{-2}$	TGV	8.6404 × 10${}^{-2}$	6.8531 × 10${}^{0}$	8.3691 × 10${}^{-1}$	185	9.0000 × 10${}^{1}$
		TV	8.7052 × 10${}^{-2}$	6.7882 × 10${}^{0}$	8.3805 × 10${}^{-1}$	212	5.0000 × 10${}^{-4}$
		TIKH	1.1476 × 10${}^{-1}$	4.3882 × 10${}^{0}$	7.4472 × 10${}^{-1}$	200	1.0000 × 10${}^{-2}$
		MULTI	**7.9139 × 10${}^{-2}$**	**7.6160 × 10${}^{0}$**	**8.5073 × 10${}^{-1}$**	4(1403)	1.0098 × 10${}^{1}$
	$10^{-2}$	TGV	6.6508 × 10${}^{-2}$	9.0670 × 10${}^{0}$	8.9245 × 10${}^{-1}$	180	2.5000 × 10${}^{2}$
		TV	6.7875 × 10${}^{-2}$	8.8903 × 10${}^{0}$	8.9272 × 10${}^{-1}$	232	1.0000 × 10${}^{-4}$
		TIKH	9.4934 × 10${}^{-2}$	5.9760 × 10${}^{0}$	8.2645 × 10${}^{-1}$	200	5.0000 × 10${}^{-3}$
		MULTI	**5.4634 × 10${}^{-2}$**	**1.0775 × 10${}^{1}$**	**9.1681 × 10${}^{-1}$**	5(1456)	1.7962 × 10${}^{0}$
	$5\times 10^{-3}$	TGV	5.3582 × 10${}^{-2}$	1.0936 × 10${}^{1}$	9.2644 × 10${}^{-1}$	292	1.0000 × 10${}^{3}$
		TV	6.2557 × 10${}^{-2}$	9.5905 × 10${}^{0}$	8.9043 × 10${}^{-1}$	283	1.0000 × 10${}^{-5}$
		TIKH	7.8021 × 10${}^{-2}$	7.6717 × 10${}^{0}$	8.2844 × 10${}^{-1}$	200	1.0000 × 10${}^{-3}$
		MULTI	**4.6590 × 10${}^{-2}$**	**1.2150 × 10${}^{1}$**	**9.4086 × 10${}^{-1}$**	7(2483)	5.8348 × 10${}^{-1}$
Gaussian	$2.5\times 10^{-2}$	TGV	7.4295 × 10${}^{-2}$	5.1521 × 10${}^{0}$	8.8489 × 10${}^{-1}$	212	5.0000 × 10${}^{1}$
		TV	7.2950 × 10${}^{-2}$	5.3107 × 10${}^{0}$	8.9032 × 10${}^{-1}$	214	5.0000 × 10${}^{-4}$
		TIKH	8.0602 × 10${}^{-2}$	4.4444 × 10${}^{0}$	8.6998 × 10${}^{-1}$	200	7.5000 × 10${}^{-3}$
		MULTI	**5.8445 × 10${}^{-2}$**	**7.2363 × 10${}^{0}$**	**9.0902 × 10${}^{-1}$**	4(650)	9.6213 × 10${}^{0}$
	$10^{-2}$	TGV	6.3055 × 10${}^{-2}$	6.4566 × 10${}^{0}$	9.1791 × 10${}^{-1}$	204	1.7500 × 10${}^{2}$
		TV	6.1079 × 10${}^{-2}$	6.7331 × 10${}^{0}$	9.3336 × 10${}^{-1}$	173	8.0000 × 10${}^{-5}$
		TIKH	6.7156 × 10${}^{-2}$	5.9092 × 10${}^{0}$	9.1910 × 10${}^{-1}$	200	2.5000 × 10${}^{-3}$
		MULTI	**4.6651 × 10${}^{-2}$**	**9.0737 × 10${}^{0}$**	**9.4174 × 10${}^{-1}$**	3(462)	1.9888 × 10${}^{0}$
	$5\times 10^{-3}$	TGV	5.6288 × 10${}^{-2}$	7.4254 × 10${}^{0}$	9.3696 × 10${}^{-1}$	222	1.0000 × 10${}^{3}$
		TV	5.7295 × 10${}^{-2}$	7.2713 × 10${}^{0}$	9.5196 × 10${}^{-1}$	155	5.0000 × 10${}^{-5}$
		TIKH	5.8965 × 10${}^{-2}$	7.0219 × 10${}^{0}$	9.3828 × 10${}^{-1}$	200	7.5000 × 10${}^{-4}$
		MULTI	**4.0354 × 10${}^{-2}$**	**1.0316 × 10${}^{1}$**	**9.5758 × 10${}^{-1}$**	4(1075)	8.1542 × 10${}^{-1}$

**Table 4:** Numerical results for the leopard test problem. Column Iters shows the number of RESPOND, ADMM, and SGP iterations for TGV, TV, and TIKH, respectively. For the MULTI algorithm, it shows the number of outer iterations and NP iterations in parenthesis. Column λ shows the used values of the regularization parameter for TIKH, TV, and TGV while, for MULTI, it reports the norm of the regularization parameters vector $\left(\lambda_{1},\ldots ,\lambda_{N}\right)$ computed by Algorithm 1. The best results are highlighted in bold.

Blur	δ	Model	RE	ISNR	MSSIM	Iters	λ
Out-of-focus	$2.5\times 10^{-2}$	TGV	1.6971 × 10${}^{-1}$	6.0610 × 10${}^{0}$	**7.5515 × 10${}^{-1}$**	221	1.2500 × 10${}^{2}$
		TV	1.7345 × 10${}^{-1}$	5.8714 × 10${}^{0}$	7.5114 × 10${}^{-1}$	276	5.0000 × 10${}^{-4}$
		TIKH	2.0715 × 10${}^{-1}$	4.3292 × 10${}^{0}$	5.8731 × 10${}^{-1}$	200	5.0000 × 10${}^{-3}$
		MULTI	**1.6854 × 10${}^{-1}$**	**6.1211 × 10${}^{0}$**	7.4807 × 10${}^{-1}$	3(325)	4.4885 × 10${}^{0}$
	$10^{-2}$	TGV	1.3874 × 10${}^{-1}$	7.7949 × 10${}^{0}$	8.0408 × 10${}^{-1}$	250	3.0000 × 10${}^{2}$
		TV	1.3360 × 10${}^{-1}$	8.1228 × 10${}^{0}$	8.0757 × 10${}^{-1}$	371	1.0000 × 10${}^{-4}$
		TIKH	1.6784 × 10${}^{-1}$	6.1411 × 10${}^{0}$	6.6137 × 10${}^{-1}$	200	1.5000 × 10${}^{-3}$
		MULTI	**1.2572 × 10${}^{-1}$**	**8.6512 × 10${}^{0}$**	**8.1534 × 10${}^{-1}$**	10(9141)	1.2627 × 10${}^{1}$
	$5\times 10^{-3}$	TGV	1.1579 × 10${}^{-1}$	9.3633 × 10${}^{0}$	**8.3657 × 10${}^{-1}$**	353	7.5000 × 10${}^{2}$
		TV	1.1976 × 10${}^{-1}$	9.0706 × 10${}^{0}$	8.2166 × 10${}^{-1}$	411	2.5000 × 10${}^{-5}$
		TIKH	1.3891 × 10${}^{-1}$	7.7821 × 10${}^{0}$	7.1537 × 10${}^{-1}$	200	5.0000 × 10${}^{-4}$
		MULTI	**1.1057 × 10${}^{-1}$**	**9.7643 × 10${}^{0}$**	8.1755 × 10${}^{-1}$	19(18607)	2.7656 × 10${}^{0}$
Gaussian	$2.5\times 10^{-2}$	TGV	1.6936 × 10${}^{-1}$	3.7549 × 10${}^{0}$	7.7259 × 10${}^{-1}$	261	1.0000 × 10${}^{2}$
		TV	1.6515 × 10${}^{-1}$	3.9736 × 10${}^{0}$	7.7529 × 10${}^{-1}$	314	4.0000 × 10${}^{-4}$
		TIKH	1.7150 × 10${}^{-1}$	3.6456 × 10${}^{0}$	6.8620 × 10${}^{-1}$	200	2.5000 × 10${}^{-3}$
		MULTI	**1.6298 × 10${}^{-1}$**	**4.0884 × 10${}^{0}$**	**7.7539 × 10${}^{-1}$**	3(241)	4.5599 × 10${}^{0}$
	$10^{-2}$	TGV	1.5058 × 10${}^{-1}$	4.7470 × 10${}^{0}$	8.0108 × 10${}^{-1}$	339	1.0000 × 10${}^{3}$
		TV	1.4747 × 10${}^{-1}$	4.9279 × 10${}^{0}$	8.0458 × 10${}^{-1}$	281	5.0000 × 10${}^{-5}$
		TIKH	1.5280 × 10${}^{-1}$	4.6194 × 10${}^{0}$	7.2992 × 10${}^{-1}$	200	5.0000 × 10${}^{-4}$
		MULTI	**1.4385 × 10${}^{-1}$**	**5.1440 × 10${}^{0}$**	**8.0937 × 10${}^{-1}$**	4(375)	1.0924 × 10${}^{0}$
	$5\times 10^{-3}$	TGV	1.4220 × 10${}^{-1}$	5.2400 × 10${}^{0}$	8.1688 × 10${}^{-1}$	500	1.0000 × 10${}^{4}$
		TV	1.4489 × 10${}^{-1}$	5.0775 × 10${}^{0}$	8.0509 × 10${}^{-1}$	309	1.0000 × 10${}^{-6}$
		TIKH	1.4156 × 10${}^{-1}$	5.2795 × 10${}^{0}$	7.7128 × 10${}^{-1}$	200	1.0000 × 10${}^{-4}$
		MULTI	**1.3314 × 10${}^{-1}$**	**5.8118 × 10${}^{0}$**	**8.2728 × 10${}^{-1}$**	5(513)	3.6611 × 10${}^{-1}$

**Table 5:** Numerical results for the elaine test problem. Column Iters shows the number of RESPOND, ADMM, and SGP iterations for TGV, TV, and TIKH, respectively. For the MULTI algorithm, it shows the number of outer iterations and NP iterations in parenthesis. Column λ shows the used values of the regularization parameter for TIKH, TV, and TGV while, for MULTI, it reports the norm of the regularization parameters vector $\left(\lambda_{1},\ldots ,\lambda_{N}\right)$ computed by Algorithm 1. The best results are highlighted in bold.

Blur	δ	Model	RE	ISNR	MSSIM	Iters	λ
Out-of-focus	$2.5\times 10^{-2}$	TGV	**5.2937 × 10${}^{-2}$**	**4.2620 × 10${}^{0}$**	7.0502 × 10${}^{-1}$	117	2.5000 × 10${}^{1}$
		TV	5.3390 × 10${}^{-2}$	4.1879 × 10${}^{0}$	7.0068 × 10${}^{-1}$	79	2.5000 × 10${}^{-3}$
		TIKH	6.7772 × 10${}^{-2}$	2.1162 × 10${}^{0}$	6.4440 × 10${}^{-1}$	200	2.5000 × 10${}^{-2}$
		MULTI	5.2967 × 10${}^{-2}$	4.2571 × 10${}^{0}$	**7.0941 × 10${}^{-1}$**	6(789)	9.2884 × 10${}^{1}$
	$10^{-2}$	TGV	4.7522 × 10${}^{-2}$	4.8898 × 10${}^{0}$	7.2933 × 10${}^{-1}$	111	1.0000 × 10${}^{2}$
		TV	4.7884 × 10${}^{-2}$	4.8238 × 10${}^{0}$	7.3036 × 10${}^{-1}$	86	5.0000 × 10${}^{-4}$
		TIKH	5.6612 × 10${}^{-2}$	3.3695 × 10${}^{0}$	6.9381 × 10${}^{-1}$	200	1.0000 × 10${}^{-2}$
		MULTI	**4.6498 × 10${}^{-2}$**	**5.0791 × 10${}^{0}$**	**7.3630 × 10${}^{-1}$**	4(426)	2.6005 × 10${}^{1}$
	$5\times 10^{-3}$	TGV	4.4345 × 10${}^{-2}$	5.4451 × 10${}^{0}$	7.4655 × 10${}^{-1}$	123	2.0000 × 10${}^{2}$
		TV	4.6262 × 10${}^{-2}$	5.0776 × 10${}^{0}$	7.4001 × 10${}^{-1}$	80	5.0000 × 10${}^{-4}$
		TIKH	5.0669 × 10${}^{-2}$	4.2873 × 10${}^{0}$	7.3213 × 10${}^{-1}$	200	5.0000 × 10${}^{-3}$
		MULTI	**4.3129 × 10${}^{-2}$**	**5.6867 × 10${}^{0}$**	**7.5707 × 10${}^{-1}$**	4(201)	5.5324 × 10${}^{0}$ >>
Gaussian	$2.5\times 10^{-2}$	TGV	4.8945 × 10${}^{-2}$	2.5540 × 10${}^{0}$	7.2618 × 10${}^{-1}$	98	1.5000 × 10${}^{1}$
		TV	4.8877 × 10${}^{-2}$	2.5660 × 10${}^{0}$	7.2428 × 10${}^{-1}$	78	2.5000 × 10${}^{-3}$
		TIKH	6.0527 × 10${}^{-2}$	7.0909 × 10${}^{-1}$	7.1350 × 10${}^{-1}$	200	3.0000 × 10${}^{-2}$
		MULTI	**4.7693 × 10${}^{-2}$**	**2.7791 × 10${}^{0}$**	**7.3403 × 10${}^{-1}$**	5(537)	8.6631 × 10${}^{1}$
	$10^{-2}$	TGV	4.5610 × 10${}^{-2}$	2.6121 × 10${}^{0}$	7.4550 × 10${}^{-1}$	100	1.0000 × 10${}^{2}$
		TV	4.5903 × 10${}^{-2}$	2.5566 × 10${}^{0}$	7.4522 × 10${}^{-1}$	75	8.0000 × 10${}^{-4}$
		TIKH	4.9219 × 10${}^{-2}$	1.9508 × 10${}^{0}$	7.3947 × 10${}^{-1}$	200	7.5000 × 10${}^{-3}$
		MULTI	**4.4332 × 10${}^{-2}$**	**2.8590 × 10${}^{0}$**	**7.5318 × 10${}^{-1}$**	3(135)	1.2558 × 10${}^{1}$
	$5\times 10^{-3}$	TGV	4.3903 × 10${}^{-2}$	2.8586 × 10${}^{0}$	7.5594 × 10${}^{-1}$	112	2.5000 × 10${}^{2}$
		TV	4.4699 × 10${}^{-2}$	2.7025 × 10${}^{0}$	7.5904 × 10${}^{-1}$	68	2.5000 × 10${}^{-4}$
		TIKH	4.6376 × 10${}^{-2}$	2.3826 × 10${}^{0}$	7.5546 × 10${}^{-1}$	200	2.5000 × 10${}^{-3}$
		MULTI	**4.2950 × 10${}^{-2}$**	**3.0493 × 10${}^{0}$**	**7.6433 × 10${}^{-1}$**	2(56)	2.9785 × 10${}^{0}$

## Data Availability

Data are contained within the article.
